# Goblet cells need some stress

**DOI:** 10.1172/JCI162030

**Published:** 2022-09-01

**Authors:** Malin E.V. Johansson, Gunnar C. Hansson

**Affiliations:** Department of Medical Biochemistry and Cell Biology, University of Gothenburg, Gothenburg, Sweden.

## Abstract

The intestinal tract is protected by epithelium-covering mucus, which is constantly renewed by goblet cells, a specialized type of epithelial cell. Mucus is largely composed of MUC2 mucin, an enormous molecule that poses a high demand on the endoplasmic reticulum (ER) for proper folding and protein assembly, creating a challenge for the secretory machinery in goblet cells. In this issue of the *JCI*, Grey et al. reveal that the ER resident protein and folding sensor ERN2 (also known as IRE1β) was instrumental for goblet cells to produce sufficient amounts of mucus to form a protective mucus layer. In the absence of ERN2, mucus production was reduced, impairing the mucus barrier, which allowed bacteria to penetrate and cause an epithelial cell stress response. This study emphasizes the importance of a controlled unfolded protein response (UPR) for goblet cell secretion.

## Mucus-producing goblet cells

Mucus is vital for the protection of epithelial surfaces in the body. It assists in numerous functions to maintain homeostasis, acting as a selective physical barrier that traps and transports unwanted components. In the colon, mucus forms a barrier both dependent on and impenetrable to bacteria, tightly linking the mucus system with the microbiota (1, 2). At the same time, mucus provides a nutritional source for the microbiota. Diseases such as ulcerative colitis (UC), in which mucus protection fails, result in epithelial cells having increased bacterial exposure (3). In cystic fibrosis, abnormally attached mucus of the small intestine leads to bacterial overgrowth (4, 5), which is similar to the phenotype observed in many lung diseases.

Mucus is produced and secreted by the highly specialized cells of the secretory lineage, the goblet cells. These cells have been considered uninteresting and homogeneous, but recent single-cell information shows dramatic variability (6, 7). Mucins are the main structural element of mucus and they, as well as some of the other abundant mucus proteins, are large proteins that are extensively disulphide stabilized, oligomeric, and highly glycosylated. The mucins are sorted and stored in large granules before being secreted. Producing large quantities of mucins is challenging for the cell and especially the endoplasmic reticulum (ER), which requires specific molecules to cope with folding and forming correct disulphide bonds. This demand makes the goblet cells specifically vulnerable to ER overloading. Healthy cells respond to biosynthetic overload through the ER stress pathway known as the unfolded protein response (UPR), comprising three branches that (a) alleviate protein misfolding, (b) reduce protein synthesis, and (c) enhance unfolded protein degradation (8). If accumulated misfolded protein amounts are large and restoration is not achieved, the cell becomes destined for apoptosis. One of the ER stress branches, as observed in most cells including enterocytes, uses the sensor ERN1 (also called IRE1α), which signals via XBP1 to induce ER-associated degradation (ERAD) and increase expression of ER proteins assisting in folding. In addition, ERAD limits translation by degrading mRNA by regulated ERN1-dependent mRNA decay. Interestingly, all goblet cells have an additional closely related molecule, ERN2 (also called IRE1β), not found in most other cells. Its specific expression indicates a distinct function in goblet cells, likely to handle mucin protein folding. Grey et al. have previously shown that ERN2 binds ERN1 to limit the ER stress responses (9). This observation indicated that ERN2 might regulate ER function in goblet cells.

## Goblet cell ER stress and inflammation

**Figure 1:**
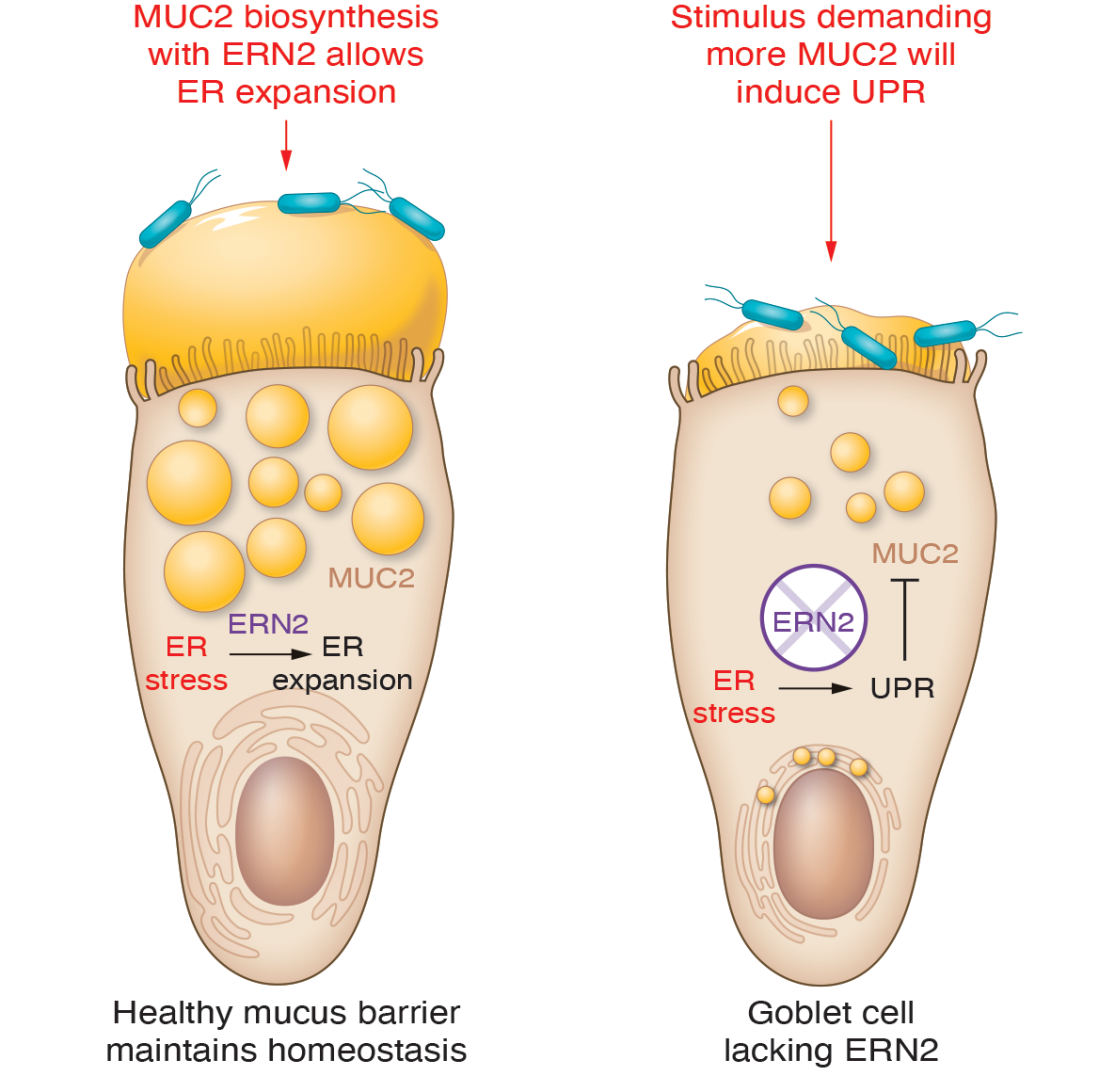
A model of ER stress response in goblet cells with and without ERN2. ERN2 is specifically expressed in goblet cells, where it allows for expansion of the ER, which is important for mucus production at homeostasis. In the absence of ERN2, goblet cells fail to produce normal amounts of mucins, resulting in ER mucin accumulation and poor protection of the epithelium. ERN2 controls and dampens the UPR. During enhanced mucus turnover and bacterial stimulation, as in colitis, ER stress and UPR responses are exaggerated and the mucus protection fails.

In this issue of the *JCI*, Grey and coauthors have continued to explore the function of ERN2 by using experimental animals (10). They nicely showed that *Ern2*-knockout mice had increased colon cell proliferation and elongated crypts as signs of mild spontaneous inflammation, which is similar to previous observations in *Muc2*-deficient mice (11). The goblet cell granule size and the number of filled goblet cells were also reduced with a diminished mucus layer (Figure 1). This finding shows that the mucus layer was less able to keep bacteria at a safe distance, triggering the epithelium to proliferate and the goblet cells to empty. The microbiota was a clear driving force for the pathology, as the germ-free *Ern2^–/–^* mice remained essentially identical to wild-type controls. The altered mucus in *Ern2^–/–^* animals also selected for less favorable microbiota that were at least partly responsible for the goblet cell and mucus phenotype observed in germ-free animals after microbiota transfer from *Ern2^–/–^* mice. Notably, this altered phenotype was reverted by wild-type microbiota, confirming the role for the microbiota in establishing a proper mucus system, as observed previously (2). As could be expected by the weaker mucus protection in the ERN2-deficient animals, these mice were more susceptible to dextran sulfate sodium–induced (DSS-induced) colitis and *Citrobacter* infection (12). In the absence of ERN2, the expression of *Xbp1s* was reduced and *Xbp1^–/–^* mice showed a phenotype similar to that of *Ern2^–/–^* mice, proving that *Xbp1* splicing induced by ERN2 was required for normal goblet cell function. Effects of the microbiota on the amount of *Xbp1* have also been observed in other studies (10, 13).

The ERN2-deficient mice resemble, in many ways, the Winnie mouse model in which misfolded MUC2 induces ER stress, as indicated by elongated crypts, fewer mucus-filled goblet cells, and increased susceptibility to induced colitis (14). Winnie mice also develop microbiota-dependent inflammation preceded by dysbiosis (15, 16). These results indicate that the mucus layer has a central role in selecting bacteria and driving epithelial and immune system responses.

## UC

Clinical pathologists often refer to UC as a goblet cell–depleting condition, since there is a decrease in the number of the typical, filled goblet cells. However, this term reflects a misunderstanding, as UC results in enhanced mucus turnover with faster goblet cell emptying and increased mucus secretion, giving the visual impression of fewer goblet cells. Defective mucus generation in *Ern2^–/–^* mice and UC patients results in a faster emptying response, especially in the upper crypt, making goblet cells not clearly visible. There is also a loss of specialized sentinel goblet cells, as these cells are ejected after TLR activation (17). Increased mucus turnover places additional pressure on the goblet cells, and accumulation of MUC2 in the ER of goblet cells is often observed in patients with active UC disease (14, 18).

## ER functions in goblet cells

The study by Grey et al. (10) shows that forming mature goblet cells capable of producing sufficient amounts of mucus requires a baseline level of UPR with increased levels of chaperones and ER expansion. This process was mediated by the goblet cell–specific ERN2 protein acting on *Xbp1*. Both ERN2 and ERN1 mediated *Xbp1* splicing (10). While ERN2 and ERN1 acted in the same pathway, they were not redundant to each other, indicating that other functions must exist, a conundrum to be studied further. It is also interesting to note that, in addition to the specific and high expression of *Ern2* in goblet cells, *Xbp1* RNA was also more abundant in goblet cells compared with other epithelial cells, while expression of *Ern1* was lower than that of *Ern2* (6). The lower endonuclease activity for ERN2 compared with ERN1 and its ability to negatively regulate ERN1 clearly indicate a different and specific mechanism for this protein (9). In addition to the known mechanisms for ERN2, including autophosphorylation and multimerization, there are likely other regulatory functions and unexplored elements.

## Conclusion

Goblet cells have a high demand on their biosynthesis machinery and require ERs with a high capacity for protein folding and modification. The current model suggests that goblet cells express specific proteins, including ERN2, to handle a required increased ER protein–folding capacity, which is similar to the ER stress response observed in other cell types, though without triggering the negative effects. The goblet cells require controlled ER stress to produce sufficient levels of mucus. Failure to produce and secrete mucus to form a protective barrier is detrimental, especially since impaired colon epithelial protection also alters the microbial milieu. In an attempt to resolve the imbalance, goblet cells enhance mucus turnover with rapid emptying. Without ERN2, the higher demand for mucus production results in undampened, enhanced ER stress (Figure 1). The specific features of goblet cells and their variability have long been ignored and warrant deeper studies. As ERN2 is an important molecule enriched in these cells, understanding its role in general and in the goblet cell–specific ER function is important for developing therapeutic approaches for inflammatory bowel diseases, especially UC.

## Version 1 09/01/2022

Electronic publication
